# Affordable passive 3D-printed prosthesis for persons with partial hand amputation

**DOI:** 10.1177/0309364620905220

**Published:** 2020-02-26

**Authors:** Raghad Alturkistani, Kavin A, Suresh Devasahayam, Raji Thomas, Esther L Colombini, Carlos A Cifuentes, Shervanthi Homer-Vanniasinkam, Helge A Wurdemann, Mehran Moazen

**Affiliations:** 1 Department of Mechanical Engineering, University College London, London, UK; 2 Christian Medical College Vellore, Vellore, India; 3 Institute of Computing, University of Campinas, Campinas, Brazil; 4 Department of Biomedical Engineering, Colombian School of Engineering Julio Garavito, Bogota, Colombia

**Keywords:** Three-dimensional printing, low-cost prosthesis, partial hand amputation

## Abstract

**Background and Aim:**

Partial hand amputations are common in developing countries and have a negative impact on patients and their families’ quality of life. The uniqueness of each partial hand amputation, coupled with the relatively high costs of prostheses, makes it challenging to provide suitable prosthetic solutions in developing countries. Current solutions often have long lead times and require a high level of expertise to produce. The aim of this study was to design and develop an affordable patient-specific partial hand prosthesis for developing countries.

**Technique:**

The prosthesis was designed for a patient with transmetacarpal amputation (i.e. three amputated fingers and partial palm). The final design was passive, controlled by the contralateral hand, and utilized the advanced flexibility properties of thermoplastic polyurethane in a glove-like design that costs approximately 20 USD to fabricate. Quantitative and qualitative tests were conducted to assess performance of the device after the patient used the final design. A qualitative assessment was performed to gather the patient’s feedback following a series of tests of grasp taxonomy. A quantitative assessment was performed through a grasp and lift test to measure the prosthesis’ maximum load capacity.

**Discussion:**

This study showed that the prosthesis enhanced the patient’s manual handling capabilities, mainly in the form of grasp stability. The prosthesis was light weight and could be donned and doffed by the patient independently. Limitations include the need to use the contralateral hand to achieve grasping and low grasp strength.

**Clinical relevance:**

Persons with partial hand amputation in developing countries lack access to affordable functional prostheses, hindering their ability to participate in the community. 3D-printed prostheses can provide a low-cost solution that is adaptable to different amputation configurations.

## Background and aim

According to the World Health Organization,^1^ about 38-million patients with amputation in developing countries lack access to appropriate prosthetic care and affordable devices. Limb loss is disproportionately high in developing countries, which account for about 2.4-million patients with upper limb amputation.^2,3^ An increasing number of these patients lose only a portion of the palm or fingers due to traumatic labour injuries and diseases, resulting in what is known as a partial hand amputation. Lack of data from developing countries makes it challenging to estimate the incidence of partial hand amputation. It is estimated that there are about 2.7-million patients with partial hand amputation in developing countries.^4^ The impact on affected patients is exacerbated by underdeveloped healthcare systems.^4^ The resultant adverse lifestyle effect can be individually devastating, and extend to the wider family if the affected patient is the main wage earner.^5^

Existing prosthetic devices for patients with partial hand amputation range from cosmetic silicone prostheses,^6,7^ to highly dexterous, mechanically actuated devices.^7^ To the best of our knowledge, these well-established passive or emerging active devices are not readily available to patients in developing countries due to the complexity, affordability and expertise required in the fabrication process.^8^ Prostheses available in developing countries have been previously reviewed extensively.^3^

Many open-source 3D-printed body-powered prostheses have been developed by researchers and designers through the Enabling The Future network (e-NABLE),^9^ and have been reviewed extensively.^10,11^ These devices are limited to patients with full transcarpal amputation. There is not yet any 3D-printed partial hand prosthesis for patients with amputation in developing countries (i.e. ones that fit patients with remaining fingers and palm) despite the advantages including short production time, low fabrication costs and simple customizability.^11^

This study uses 3D-printing techniques to develop an affordable partial hand prosthesis for patients with amputation in developing countries made from flexible material. We describe the evolution of our design process and the experimental results with a patient in the Rehabilitation Institute of Christian Medical College Hospital, Vellore, India. The final device can be donned and doffed independently, is light weight, adds stability to the grasp and is passively controlled by the contralateral hand to produce the desired grasp. Note that the uniqueness of this hand is not in its actuation mechanism, but rather in (1) its flexible material and glove-like, comfortable fit that is not provided by typical ‘clamping’ prostheses and (2) its light weight and compactness. Those factors combined with the advantages of 3D printing (simple customizability, low-cost and short production time) provide a unique solution for persons with partial hand amputation who retain the majority of their hand’s function and to whom comfort and weight are the most critical factors when choosing a prosthesis.

## Technique

### Patient

**Figure 1.**
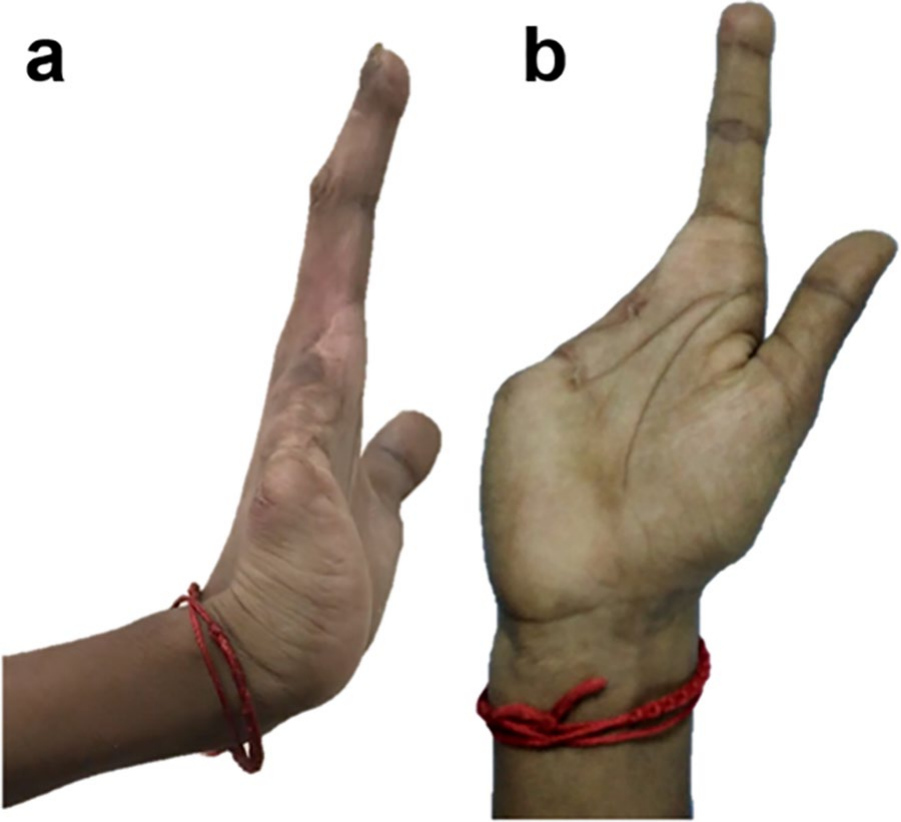
(a) Side view and (b) palmar view of the patient’s right hand showing the amputation wherein the middle, ring, little finger and a distal portion of the palm are missing.

Ethical approval was obtained for this study at the Christian Medical College (CMC) in Vellore, India. Furthermore, written informed consent was obtained for patient information and images to be used in a publication. The recruited male patient was 1.75-m tall and weighed 49 kg. The patient experienced a traumatic labour injury resulting in a transmetacarpal amputation, that is, missing three fingers (middle, ring and little finger) of the dominant (right) hand and a distal portion of the palm as shown in Figure 1. The patient had rejected any cosmetic prosthesis and only used a cloth wrapped around his partially amputated right hand as a cosmetic cover and to enhance grip.

### Design process

Table 1 summarizes the design evolution of various prostheses prototypes for the recruited patient. The initial designs were based on dimensioned drawings of the patient’s hand, followed by hand moulds and a 3D scan. The models were developed using Autodesk Fusion 360 (Autodesk, Inc., San Rafael, CA, USA) and 3D printed on different desktop printers. The initial design was based on e-NABLE’s Raptor Reloaded hand, a 2-part wrist-powered polylactic acid (PLA) device.^9^ However, due to the device’s size and restriction of movement (Table 1), the palmar and wrist parts were modified in subsequent prototypes to make the device smaller and more flexible to better fulfil the needs of a transmetacarpal amputation. For simplicity, the prosthetic fingers remained unchanged, using the same design as that of e-NABLE’s Raptor Reloaded hand.^9^ The design evolved from using active wrist-power to a passive prosthesis based on the patient’s feedback during the design process.

**Table 1.** Design evolution and display of various prototypes made out of PLA and TPU.

**Prototype**	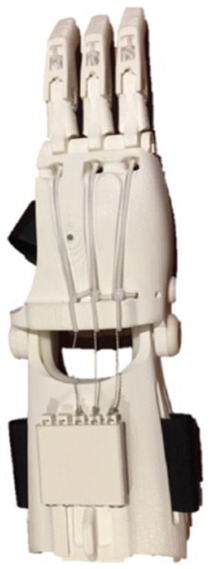	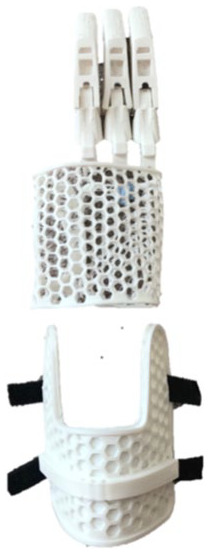	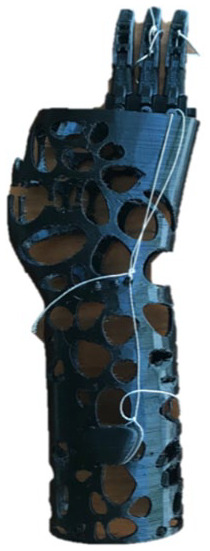	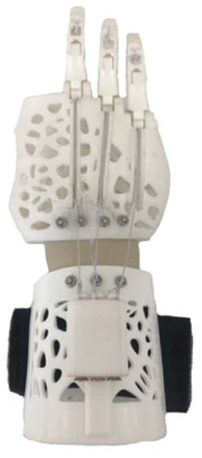	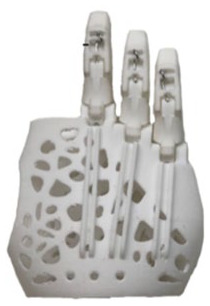
**Design description**	2-part wrist-powered, based on e-NABLE’s Raptor Reloaded hand modified to the amputee’s configuration. (24 USD)	2-part active wrist-powered. Printed flat and thermoformed to accurately fit the patient (0 USD).	1-part flexible design with palm filler, organically shaped for a glove-like prosthesis (27 USD).	2-part active wrist-powered design. Organic glove-like palm and thermoformed wrist (23 USD).	1-part passive organic glove-like design. Extremely compact and lightweight (20 USD)
**Main body material**	PLA	PLA	Ninjatek Ninjaflex® (Fenner Drives, Inc., Manheim, PA, USA)	Ninjatek Ninjaflex (palm), PLA (wrist)	Ultimaker TPU (Ultimaker, Geldermalsen, The Netherlands)
**Printer**	Ultimaker 2	Ultimaker 2	Replicator 2 (Makerbot, Brooklyn, NY, USA)	Ultimaker 2, Replicator 2	Ultimaker 2
**Disadvantages**	• Bulkiness • Restriction to wrist movement	• Fragility • Discomfort due to inorganic shape	• Fragility • Long print hours	• Length of the device • Abundance of mechanical parts	• Unsecure fit • Minor restriction to movement

PLA: polylactic acid; TPU: thermoplastic polyurethane.

### Final design

**Figure 2.**
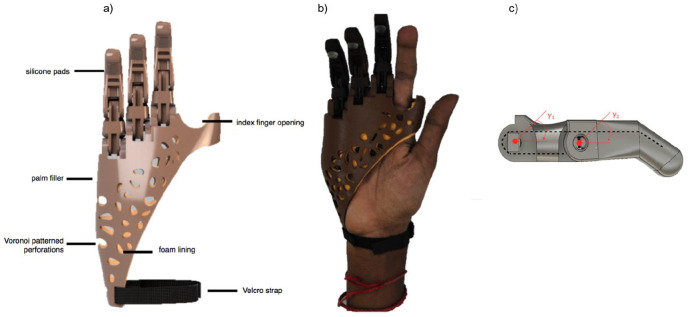
(a) Dorsal view of the rendering of the final produced design, (b) the patient wearing the final prototype and (c) an illustration of the fingers’ degrees of freedom. The thumb and finger openings were enlarged to enable more freedom of movement. Fingers were printed using polylactic acid (PLA) while the palmar part used thermoplastic polyurethane (TPU). Fingers have a galvanized wire extended within them (dashed line).

Figure 2(a) shows the final prototype consisting of a passive 3D-printed device that extended only to the wrist to maintain its degrees of freedom. The device was customized to the patient’s hand anatomy and therefore had two openings for the residual index finger and thumb. Three prosthetic fingers were mounted on the palm section of the prosthesis and filler substitutes for the missing volume of the palm. Individual pieces of a 1.2-mm galvanized wire were passed through and wrapped around the fingers’ internal openings as indicated in Figure 2(c). The lengths of those wires were manipulated to enable desired grasp configurations. Voronoi-patterned perforations provided ventilation to the patient’s hand for added comfort. This pattern can be automatically generated, making it simpler to reproduce. The final prototype weighed less than 100 g and cost less than 20 USD to fabricate. The compact glove-like design simplified independent donning and doffing by the patient.

The palmar part of the device was printed using thermoplastic polyurethane (TPU) of 85A shore hardness and 1.75-mm diameter on the 3D printer Prusa i3 MK3 (Prusa Research S.R.O., Prague, Czech Republic). The fingers were printed using PLA (1.75 mm) by an Ultimaker 2 printer (Ultimaker BV, Utrecht, The Netherlands). Other components included a medical-grade 2-mm polyethylene foam sheet to provide softer padding and sweat-absorption, a Velcro strap to provide an adjustable, tight fit and 3M self-adhesive silicone pads to provide a better grip to the fingertips.

The primary function of this partial hand prosthesis was to provide a stable grip to improve the ability to perform bimanual and unilateral activities. Figure 2(b) shows the fingers’ degrees of freedom. When working with this device, the patient used the contralateral hand to position the prosthetic fingers to the desired grasp configuration, which then stayed in place through the galvanized wires that have been tensioned to enable such configurations.

### 3D-printing specifications

For the palmar part, the printer was set to 42 mm/s print speed, 75% infill, 0.2-mm layer height and 240°C extruder temperature. The fingers were printed with 70-mm/s print speed, 20% infill, 0.2-mm layer height and 205°C extruder temperature.

### Quantitative and qualitative assessment

Performance of the devices was assessed through a series of quantitative and qualitative tests. Qualitative assessment was conducted using open-ended questions administered after the patient put on the final design of the prosthetic hand and performed a series of tests based on the grasp taxonomy.^11,12^

**Figure 3.**
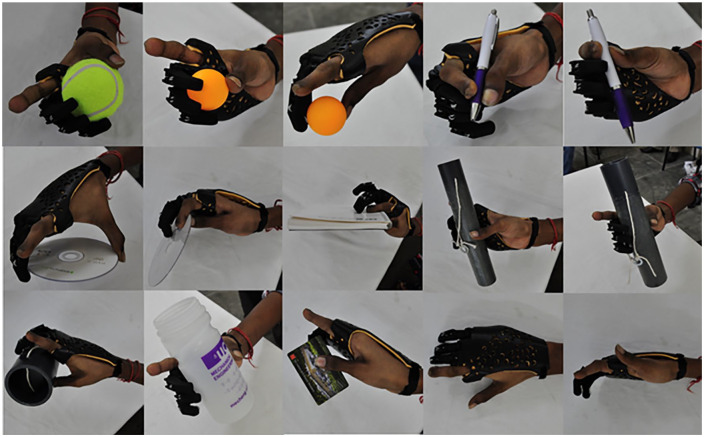
Demonstration of the grasp taxonomy test.^13^ The grasps were divided into power and precision grasps. The use of index and thumb were minimized to ensure testing of the prosthesis itself.

The grasp taxonomy test performed in this study was based on the approach described by Feix et al.^13^ as conducted by Sayuk.^12^ Since the patient in this case had an index and thumb, grasps that rely only on these two fingers were eliminated. The patient was asked to attempt to grasp each object using the prosthetic hand (with the aid of the contralateral hand when needed). The patient was asked to eliminate use of the index finger (in the amputated hand). This process was repeated 3 times, and the results are shown in Table 2. Using the prosthesis, the patient was able to perform 12 out of 14 grasps, one of them being approximate. All grasps were possible without the prosthesis, however, with less stability (i.e. deficiency of ability or control) to the grasp. Figure 3 shows pictures of the patient demonstrating each of the possible grasps in the list.

**Table 2.** Results from the grasp taxonomy test based on the grasp taxonomy matrix.^13^

Object	Grasp no. as in ten Kate et al.^11^	Grasp type	Prosthesis	No prosthesis
Tennis ball	26	Power	Possible	Possible
Ping pong ball	14	Precision	Possible	Possible
Ping pong ball	27	Precision	Possible	Possible
Arbitrary object	12	Precision	Possible	Possible
Pen	6	Precision	Possible	Possible
Pen	20	Precision	Possible	Possible
CD 1	10	Power	Approximate	Not possible
CD 2	18	Precision	Not possible	Possible
Notebook	22	Precision	Possible	Possible
Scissors	19	Power	Not possible	Approximate
Pipe 1	2	Power	Possible	Possible
Pipe 2	15	Power	Possible	Possible
Pipe 3	1	Power	Possible	Possible
Card	16	Precision	Possible	Possible

The second test conducted was the grasp and lift test that measured the prosthesis’ payload capabilities by lifting an object of a certain weight, increasing the load gradually and measuring the maximum load that could be lifted.^14^ The object’s shape and size were limited to a cylindrical shape of diameter 50 mm using a cylindrical wrap grasp that maximized the number of object-hand contact points. The test results show that the maximum weight that could be lifted and held for a duration of 3 s by the prosthesis was 700 g.

## Discussion

The production of affordable, functional partial hand prostheses is challenging due to the unique anatomy of each patient with amputation. In this study, 3D design and printing technologies were utilized to produce an affordable, passive partial hand prosthesis that may be customized for different amputation configurations.

Feedback from the patient indicated that the main advantages of the device were its compactness, light weight, and simple donning and doffing. When presented with a similar design that provided active function and extended beyond the wrist (design 1, Table 1), the patient preferred the passive and compact design. Despite low grip strength, the device’s function was sufficient for this patient’s case as his vital need was the provision of a stable grasp through substitution of the missing part of the palm. The patient preferred the device over the cosmetic devices available at the local medical centre and the no-prosthesis option. This was because the integrated filler improved stability of different grasps and the patient’s ability to perform some bimanual activities, which he believed would enable him to perform his job better.

The produced device costs less than 20 USD, making it 200% cheaper than silicone-based devices to fabricate.^6^ It is one third the weight of a traditional prosthesis and can be manufactured in 1 day.^7^ This provides a more efficient alternative for use in developing countries that experience a shortage of trained personnel as the production process is less labour-intensive.^3,15^ 3D-scanning and computer-aided design packages simplify the process of accurately sizing and resizing the device to the individual’s residual limb.

Considering the design evolution of various prototypes developed in this study, printing the palmar portion of the device using TPU increased its comfort. This was due to the increased flexibility of the prosthesis compared to printing it in more rigid materials such as acrylonitrile butadiene styrene (ABS). Although TPU has a higher cost than PLA, the compact design of the produced device uses less material than the PLA 3D-printed prosthesis, reducing the overall cost (see Table 1).

A limitation to this device is that the grips are achieved using the contralateral hand and is maintained with the galvanized wire. While this may allow the device to be more practical, it contributes to its low grip strength (700 g hold limit). This could possibly be overcome using alternative, more durable materials for the fingers; thicker galvanized wire; and increasing the friction between interlocking finger parts. Rapid improvements in the field of 3D printing suggest that issues like durability and design parametrization may soon be overcome, yet in the current state, 3D printing seems to be a highly effective alternative to traditional methods of prostheses fabrication in developing countries.

## Key points

- Developed a passive and flexible 3D-printed partial hand prosthesis.
- Significantly reduced lead times compared to traditional prostheses.
- Significantly reduced costs compared to 3D-printed and other prostheses.
- A more appropriate prosthetic solution for developing countries.
